# Noise-induced bistability in the fate of cancer phenotypic quasispecies: a bit-strings approach

**DOI:** 10.1038/s41598-018-19552-2

**Published:** 2018-01-18

**Authors:** Josep Sardanyés, Tomás Alarcón

**Affiliations:** 1 Centre de Recerca Matemàtica, Campus de Bellaterra, Edifici C, 08193 Bellaterra, Barcelona, Spain; 2 Barcelona Graduate School of Mathematics (BGSMath). Campus de Bellaterra, Edifici C, 08193 Bellaterra, Barcelona, Spain; 3 ICREA, Pg. Lluis Companys 23, 08010 Barcelona, Spain; 4 Departament de Matemàtiques, Universitat Autònoma de Barcelona, Barcelona, Spain

## Abstract

Tumor cell populations are highly heterogeneous. Such heterogeneity, both at genotypic and phenotypic levels, is a key feature during tumorigenesis. How to investigate the impact of this heterogeneity in the dynamics of tumors cells becomes an important issue. Here we explore a stochastic model describing the competition dynamics between a pool of heterogeneous cancer cells with distinct phenotypes and healthy cells. This model is used to explore the role of demographic fluctuations on the transitions involving tumor clearance. Our results show that for large population sizes, when demographic fluctuations are negligible, there exists a sharp transition responsible for tumor cells extinction at increasing tumor cells’ mutation rates. This result is consistent with a mean field model developed for the same system. The mean field model reveals only monostability scenarios, in which either the dominance of the tumor cells or the dominance of the healthy cells is found. Interestingly, the stochastic model shows that for small population sizes the monostability behavior disappears, involving the presence of noise-induced bistability. The impact of the initial populations of cells in the fate of the cell populations is investigated, as well as the transient times towards the healthy and the cancer states.

## Introduction

Cancer progression is known to involve the emergence of highly heterogeneous populations of tumor cells. This is a defining feature of most advanced tumors^1,2^. Despite this seeming heterogeneity, tumor cells share some common traits that have been labeled as the hallmarks of cancer^3,4^. These hallmarks include self-sufficiency in growth signals, insensitivity to anti-growth signals, evasion of apoptosis, and limitless replicative potential. Typically, this acquired characteristics lead to an abnormal increase in cells’ proliferation rates. Also, tumor cells can usually sustain angiogenesis and, in mid or late stages, invade other tissues and metastasize. Other universal traits have been proposed as hallmarks of cancer^4^: the deregulation of cell energetics, avoiding immune system, tumor-promoting inflammation, and genome instability and mutation.

Acquisition of these multiple hallmarks depends largely on a succession of alterations in the genomes of neoplastic cells^4^. Roughly speaking, certain mutant genotypes confer selective advantage on subclones of cells, enabling their outgrowth and an eventual dominance in a given local environment^4^. Interestingly, the so-called heritable phenotypes do not restrict the increase in heterogeneity and diversity to genotypic changes. For example, activation of tumor suppressor genes can be acquired through epigenetic changes such as DNA methylation and histone modifications^5–7^. However, genome instability is known to play a key role in the evolutionary dynamics of cancer. Genome instability refers to an increased tendency to accumulate alterations and mutations in the genome during the life cycle of tumor cells. It is known that healthy cells have a mutation rate of about 1.4 × 10^−10^ changes per nucleotide and replication cycle^8^. It has been proposed that the spontaneous mutation rate of normal cells is not sufficient to account for the large number of mutations found in human cancers. Indeed, studies of mutation frequencies in microbial populations, in both experimentally-induced stress and clinical cases, reveal that mutations that inactivate mismatch repair genes result in 10^2^−10^3^ times the background mutation rate, with comparable increases in cancer cells^9–16^. Moreover, the accumulation of mutations can be accelerated by compromising the surveillance systems that normally monitor genomic integrity and force genetically damaged cells into either senescence or apoptosis routes^17–19^, where the role of TP53 is central^20^.

The presence of high genome instability creates a peculiar situation: since cancer cells typically become less differentiated, more capable to adapt but also more prone to failure, how much instability can be afforded by cancer cells? It has been suggested that unstable cancer progression may be feasible up to some critical instability levels^21–27^. Once this critical point is reached, instability levels shall become lethal. Such evolution towards the edge of instability has been reported for RNA viruses, where mutagenic thresholds have been found^28–30^. RNA viruses have been suggested to exhibit critical mutation rates^29,31^, beyond which they could experience extinction. RNA viruses replicate at extremely large mutation rates^32^, and they are characterized by a highly heterogeneous population of close-related genomes known as *quasispecies*^32–34^. Due to the largely increased mutation rate of tumor cells, mainly driven by genome instability, the quasispecies concept has been also applied to cancer evolution^21–23,25,35,36^. Following these similarities, and due to increased genome instability, it has been suggested that cancer cells could also present critical instability thresholds^21,22,26,27,36^. If true, cancer treatments could incorporate increased mutagenesis that would push the system slightly beyond its critical mutation limits^12^. Hence, a possible therapeutic strategy in tumors would benefit from targeting DNA repair pathways^37–40^. Similarly, germline mutations in the proofreading domains of DNA polymerases Pol *δ* and *ε* have been identified in many types of cancers, giving place to the so-called ‘ultramutator’ phenotype^41^. It has been suggested that increased mutation in these tumors (e.g., breast cancer, colorectal cancer, or anaplastic astrocytoma, to cite some) with impaired exonuclease proof-reading activities could drive cancer towards such hypothetical critical transitions^41^. Recent research on critical transitions has focused on how to predict critical thresholds as a way to provide early detection of diseases such as cancer^42^. For instance, shift predictions between different phenotypic states due to stochastic gene expression^43^.

Theoretical models on cancer quasispecies have revealed continuous, smooth transitions towards the collapse or the impairment of tumor cells, mainly governed by transcritical bifurcations as some of the model parameters (typically mutation rates) are tuned^21,22,36^. By smooth transition we mean that the population equilibria decrease monotonously until its extinction as a given parameter is continuously changed. These models usually deal with mutation rates as single parameters that can be increased to explore their impact on tumor cells’ fate. However, a more realistic approach should include a repertoire of changes affecting not only genome stability but also replication traits (e.g., mutations or anomalies in tumor suppressor genes and proto-oncogenes). Similarly, the limits imposed to instability levels are largely determined by the presence of essential (house-keeping, hereafter *hk*) genes, whose integrity needs to be preserved^25^. Following this rationale, a digital genomes model incorporating multiple genes responsible for replication and stability, as well as the use of *hk* genes, suggested that a complex evolution unfolds as the cancer population approaches higher, near-critical instability levels^24,25^. Despite these recent computational studies on the topological characteristics of evolutionary cancer networks, the dynamics and bifurcations governing the population dynamics of healthy cells competing with a heterogeneous pool of cancer phenotypes^35^ remain poorly understood, especially the impact of demographic noise due to small population sizes, expected to occur at the beginning of the tumor progression progress, where a tiny number of anomalous cells can initiate this process in small tissues.

The impact of stochastic fluctuations in cancer growth and evolution has been studied with diligence in the last decades. Stochasticity in cancer growth can arise from many different sources. As mentioned, demographic (intrinsic) noise due to finite-size populations can play a key role at the initial stages of tumorigenesis. Other extrinsic sources of noise affecting the complexity of tumorigenesis can be given by random variations in nutrients supply or in the availability of oxygen, among others. Stochastic evolutionary dynamics has been explored for leukemic cells under different targeted therapies using MonteCarlo simulations^44^. Also, the Langevin equation has been employed to characterize stochastic cancer evolution for chronic myeloid leukemia^45^. This equation has been used to study the impact of noise in a model for cancer growth considering the cytotoxic response of the immune response^46^. Here, the presence of resonant activation and noise-enhanced stabilization, NES, was identified (see also ref. ^47^ and the Conclusions Section for a wider discussion on NES).

Here we explore the impact of demographic fluctuations in a dynamical system formed by a population of healthy cells competing with a heterogeneous population of distinct tumor cell phenotypes. These phenotypes include cell populations with increased proliferation rates due to e.g., mutations or anomalies in tumor suppressor genes (e.g., TP53 or APC) and in proto-oncogenes (such as RAS or SRC). Although they act in different ways, here we make no explicit distinction since anomalies in both gene types involve an increased proliferation of tumor cells^3,4,48^. Moreover, tumor cells can also undergo increased genome instability due to mutations or anomalies in genes preserving genomic integrity (such as BRCA-1, BLM, ATM, or TP53). This dynamical system is built using a bit-strings approach, where cells phenotypic traits are coded in 3-bits sequences. The first bit correspond to the compartment of replication-related genes (tumor suppressor genes and proto-oncogenes); the second bit to the compartment of genome instability-related genes; and a third compartment (third bit) corresponding to the *hk* genes compartment. The *hk* genes are essential for the survival of cells and their failure leads to cell death. The *hk* genes are constantly expressed in cells and would include, for example, ubiquitin, GAPDH, or ribosomal proteins, among others^49^.

Our main goal is to investigate the scenarios of dominance of either tumor or healthy cells evaluating the impact of demographic noise by using a phenotypic quasispecies model. The phenotypic quasispecies framework has been recently used to investigate the effects of mutational fitness effects in RNA viral populations by means of ordinary differential equations (ODEs)^50^. A mean field model on cancer phenotypic quasispecies considering the above mentioned phenotypes competing with healthy cells has been recently studied^51^. This model revealed the presence of a catastrophic extinction of tumor cells at increasing the mutation rate of tumor cells. In contrast to a smooth transition, a catastrophic one involves a discontinuous change of the population equilibria (e.g. a change from survival to extinction) as the control parameter is continuously varied. This transition was given by the so-called trans-heteroclinic bifurcation (see refs^51,52^. for a description of this bifurcation). As a difference from the ODEs model, which considers a continuum in population numbers and determinism, we here use an agent-based MonteCarlo model, which considers finite populations and stochasticity. Previous theoretical works on stochastic Boolean genetic elements in cancer gene regulation reveal the importance of noise in the emergence of noise-induced multistability^53^. Here we are especially interested in the nature of the transition separating tumor cells persistence from their extinction under stochasticity, as well as in the role of the initial populations in the fate of the cells and in the transient times towards both tumor and healthy states.

## Results

### Summary of the mean field dynamics

The dynamical system explored in this article has been recently investigated by means of a mean field model given by ordinary differential equations (ODEs)^51^. This mathematical model was developed from the well-known Eigen’s quasispecies equation, within the framework of the so-called phenotypic quasispecies^50,51^. Here, as a difference from the standard quasispecies model^33^, the sequences are used to code for the phenotypic traits of the replicators (see Supplementary Table S1) instead of being used to describe the dynamics of information of RNA or DNA genomes explicitly. The ODEs model on cancer phenotypic quasispecies identified two qualitatively different dynamical outcomes (see Figs S1b and S2): (i) outcompetition of the healthy cells by the tumor cell populations; and (ii) outcompetition of the tumor cell phenotypes by the healthy cells. The transition from state (i) to state (ii) is governed by a global bifurcation, the *trans-heteroclinic* bifurcation (see refs^51,52^. for details on this bifurcation).

To summarize, the trans-heteroclinic bifurcation is a global bifurcation that involves an exchange of stability between two fixed points without their collision^52^, as opposed to the transcritical bifurcation. Below the trans-heteroclinic critical value, there exists a stable fixed point that is connected with an unstable one by a heteroclinic connection (see Definition I.1 in the SM). At the bifurcation point, the heteroclinic connection is replaced by a line of fixed points. Above the bifurcation threshold, there is an exchange of stability between the two fixed points present before the bifurcation, and the heteroclinic connection is recovered^51^. The mean field model revealed that key parameters responsible for the bifurcation were: *r* (cells’ replication rates), *δ*_*r*_ (increase in proliferation rates in tumor cells), and *μ* (rate of accumulation of mutationas or genome anomalies). Specifically, the bifurcation value was given by:

1 $${\mu }_{c}={\delta }_{r}/(r+{\delta }_{r}\mathrm{).}$$

With *μ* < *μ*_*c*_, a fixed point corresponding to the extinction of healthy cells and the dominance of some of the tumor cell phenotypes (named ${P}_{3}^{\ast }$ in^51^) was globally asymptotically stable, while the fixed point corresponding to the dominance of the healthy cell populations and the extinction of all of the tumor cell phenotypes (healthy state, with equilibrium labeled ${P}_{2}^{\ast }$ in^51^) was unstable. With *μ* > *μ*_*c*_ the two fixed points interchanged the stability, ${P}_{3}^{\ast }$ becoming unstable and ${P}_{2}^{\ast }$ being globally asymptotically stable (see Fig. S2). That is, under the bifurcation scenario described in^51^, monostability was found i.e., there was no possibility for both fixed points ${P}_{2}^{\ast }$ and ${P}_{3}^{\ast }$ to be stable under the same parameter values. We refer the reader to Section SI.A. in the Supplementary Material for further details on the results obtained from the mean field model^51^.

### Catastrophic extinction of tumor phenotypes

**Figure 1:**
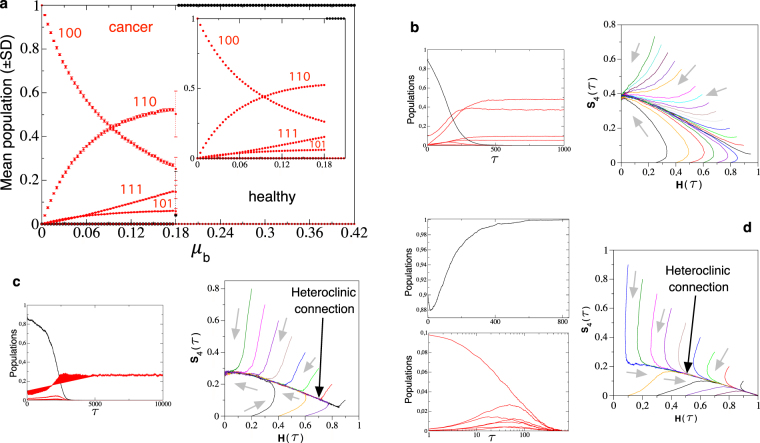
(**a**) Mean population equilibria obtained from the stochastic bit-strings model using large population sizes (*N* = 5 × 10^4^ cells in the main panel; *N* = 10^5^ cells in the inset). All data points are the mean population values (±*SD*) computed at *τ* = 25000 generations averaged over 25 independent replicas (tumor phenotypes and healthy cells are plotted using red and black dots, respectively). We also display time series and the dynamics projected in the simplex Ω for different values of *μ*_*b*_ below, close to, and above the critical per-bit mutation rate using a population of *N* = 10^5^ cells. Specifically we use: (**b**) *μ*_*b*_ = 0.12; (**c**) *μ*_*b*_ = 0.18; (**d**) *μ*_*b*_ = 0.22. The red and black time series correspond to the tumor and healthy phenotypes, respectively. The gray arrows inside the simplexes indicate the direction of the trajectories. In all of the analyses the probabilities are fixed to the same values used in Fig. S2: *r* = 0.1, ${\delta }_{r}={\delta }_{{\mu }_{b}}=0.05$. Notice that the heteroclinic connection identified with the ODEs model studied in^51^ is also found in the bit-strings stochastic model (see the projections of the stochastic trajectories in (**b**) and (**c**), where the connection can be clearly visualized. See also Fig. S2).

Before focusing on the impact of demographic noise in the dynamics of the system studied in this article, we will explore its dynamics for large population sizes (large *N*), thus mimicking the mean field model. The bifurcation identified in ref.^51^, named trans-heteroclinic, which caused a catastrophic extinction of tumor cell populations (see Fig. S1b) is also obtained from the stochastic bit-strings model for large *N* (see the Methods Section for the description of the simulation model). Figure 1 displays the mean population of each phenotype at increasing *μ*_*b*_. Specifically, the main panel displays the mean population values for all cell states of the quasispecies setting *N* = 5 × 10^4^ cells. Notice that the mean population values and their response to the increase in *μ*_*b*_ perfectly match with the results obtained from the mean field model (compare the bifurcation diagram of Fig. 1 with the one displayed in Fig. S1b). The trans-heteroclinic bifurcation can be perfectly identified in the inset of the bifurcation diagram in Fig. 1, obtained with *N* = 10^5^ cells. In Fig. S1b and S2 we have set *r* = 0.1 and ${\delta }_{r}={\delta }_{{\mu }_{b}}=0.05$, thus using the same values for these parameters used to obtain the bifurcation diagram of Fig. 1b. The critical value of *μ*_*b*_ obtained from the simulation model is ${\mu }_{b}^{c}\approx 0.18$.

The bifurcation value provided by equation (1) corresponds to an overall mutation rate for the tumor cells. In our simulation model we are considering the per-bit mutation rate, *μ*_*b*_, thus in order to compare both bifurcation values we must compute a mean mutation probability for the entire string from the mutation probability for each position, given by *μ*_*b*_. Let us define the average mutation probability in our model as $\hat{{\mu }}=1-\varepsilon$, with $\hat{{\mu }}\in \mathrm{[0},\mathrm{1]}$, and *ε* ∈ [0, 1] being the probability that the entire sequence will be replicated accurately. Such a probability in our system can be approximated (considering ${\delta }_{\mu }\ll 1$) by *ε* = (1 − *μ*_*b*_)^*ν*^, with *ν* = 3, *ν* being the length of the sequence (number of bits). For the parameter values *r* = 0.1 and *δ*_*r*_ = 0.05, the critical mutation rate for the mean field model given by equation (1) is *μ*_*c*_ = 1/3. The critical per-bit mutation value, ${\mu }_{b}^{c}$, obtained from the critical average mutation rate ${\mu }_{c}={\hat{{\mu }}}_{c}=\frac{1}{3}=1-{\mathrm{(1}-{\mu }_{b}^{c})}^{3}$ is given by ${\mu }_{b}^{c}\approx 0.13$, which approaches the value ${\mu }_{b}^{c}\approx 0.18$ obtained from the simulations.

The analyses performed with the mean field model (see^51^ and Section S1 in the Supplementary Material, SM) revealed that the fixed points ${P}_{2}^{\ast }$ and ${P}_{3}^{\ast }$ presented a heteroclinic connection (see Fig. S2). The definition of a heteroclinic connection can be found in Definition I.1 in the SM.

Interestingly, the simulations performed with the bit-strings model for large *N* clearly reveal the presence of this heteroclinic connection (indicated with a black arrow in panels (c,d) of Fig. 1). For example, the projections of the trajectories in the simplexes (*H*,*S*_4_) in panels (b–d) of Fig. 1 show that the trajectories travel towards this connection and then achieve one of the two possible equilibria, either the tumor state *T* (Fig. 1(c)) or the healthy state *H* (Fig. 1(d)). For the sake of clarity compare these projections with the ones displayed in Fig. S2 below and above the trans-heteroclinic bifurcation. Finally, in Fig. 1 we also display some time series for each of the three values of *μ*_*b*_ analyzed in panels (b–d).

### Impact of noise on the trans-heteroclinic transition

**Figure 2:**
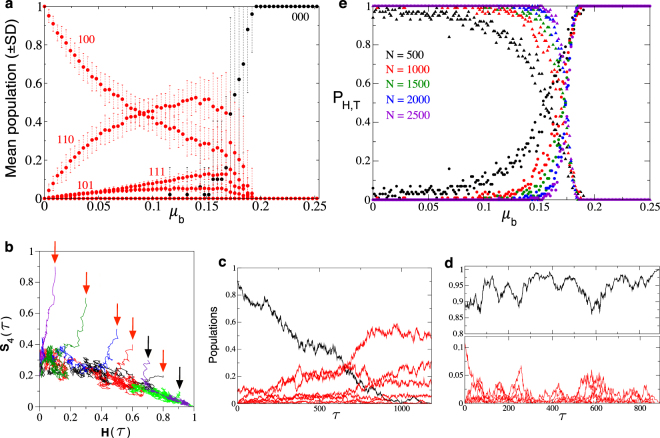
(**a**) Mean population equilibria setting *N* = 500 cells. Each data point is the mean (±SD) population computed at *τ* = 25000 generations averaged over 25 independent replicas. (**b**) Stochastic trajectories projected in the space (*H*(*τ*), *S*_4_(*τ*)) setting *μ*_*b*_ = 0.18. Notice that here the dynamics takes place on the heteroclinic connection. The trajectories indicated with the black and red arrows achieve, respectively, the healthy (*H*^*as*^) and the tumor (*T*^*as*^) absorbing states. (**c**) Stochastic time series of healthy (black) and tumor (red) cells also using *μ*_*b*_ = 0.18. (**d**) Time serie also with *μ*_*b*_ = 0.18 using the same initial populations as in (**c**). We note that here the asymptotic state changes, and the population of healthy cells outcompetes the population of tumor cells. (**e**) Probability, *P*_*H*,*T*_, of achieving the healthy *H*^*as*^ and the tumor *T*^*as*^ absorbing states within the same range of mutation displayed in (**a**). Each data point is the value of *P*_*H*_ (circles) and *P*_*T*_ (triangles) computed from 100 replicates. Here five different population sizes have been simulated. In panels (**a**) and (**c**–**e**) we have used an initial population of 450 healthy cells and 50 tumor cells with sequence 100.

In the following sections we will explore the impact of demographic noise on the dynamics of both healthy cells and tumor cell phenotypes by using small population sizes, focusing on the transition given by the trans-heteroclinic bifurcation. Figure 2a shows the same bifurcation diagram displayed in Fig. 1, now computed with *N* = 500 cells. Notice that the mean population values are kept similar to the ones displayed in the previous diagrams (see Figs 1 and S1b) but now the standard deviation in the mean population values increases, especially when *μ*_*b*_ approaches its critical value. This means that the abrupt and sharp transition identified with the ODEs model in ref.^51^ as well as in the simulations setting *N* large is replaced by a smooth transition. This phenomenon is clearly induced by demographic fluctuations, since the monostability character identified with the ODEs model is lost. Hence, we have identified a new phenomenon for trans-heteroclinic bifurcations under noise: monostability is replaced by bistability, which is noise-induced. Stochasticity can make trajectories to reach either the tumor or the healthy absorbing states under the same combination of probabilities for replication and mutation. An example of the loss of monostability is illustrated in Fig. 2(b), where the same projection on the simplex Ω displayed in Fig. S2 and Fig. S1b is shown. Here seven stochastic trajectories are plotted in the space (*H*(*τ*), *S*_4_(*τ*)). Five of these trajectories reach the tumor state, while two of them achieve the healthy state. The panels (c) and (d) in Fig. 2 display time series for each of these two qualitatively different scenarios (panel c: tumor state; panel d: healthy state). Notice that trajectories display strong fluctuations due to the small population size used in these simulations.

We want to note that the use of the mean populations of cells as an order parameter is not the most appropriate way to investigate the transition given by the trans-heteroclinic bifurcation with stochastic fluctuations, especially for small population sizes and when the control parameter approaches the phase transition (bifurcation) value. Since the system is monostable in its infinite-diffusion, deterministic limit, the equilibrium population values for healthy cells correspond to zero population or to a population dominated by tumor cells^51^. Moreover, the equilibrium populations of tumor cells below the trans-heteroclinic bifurcation were shown to depend on the replication and mutation parameters^51^.

In order to characterize the nature of the phase transition arising in the stochastic model better we will use the probability of achieving a given absorbing state, either the healthy *H*^*as*^ or the tumor *T*^*as*^ one, given by *P*_*H*_ and *P*_*T*_, respectively (which can be computed in different ways, see below). The behavior of this order parameter upon the increase of *μ*_*b*_ is displayed in Fig. 2(e) for five small population sizes. Here, *P*_*H*_ has been computed by dividing the number of simulations achieving *H*^*as*^ by the total number of runs (we have used 100 replicates) starting with initial condition (*H*(*τ* = 0), *S*_4_(*τ* = 0)) = (0.9, 0.1). Notice that as *N* increases, the sigmoidal-like shape of the data approach a step function, which is obtained for very large population values (e.g., *N* = 5 × 10^4^ or *N* = 10^5^ cells, see Fig. 1). Specifically, we plot the values of *P*_*H*_ (circles) and *P*_*T*_ (triangles). Hereafter we will restrict our computations to *P*_*H*_, since *P*_*T*_ = 1 − *P*_*H*_.

**Figure 3:**
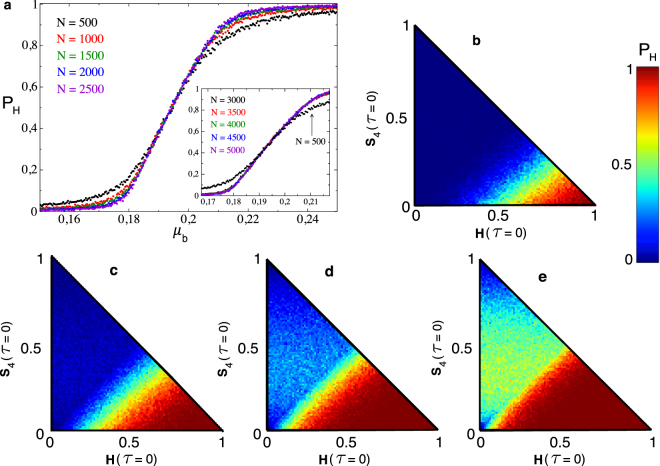
(**a**) Probability of achieving the healthy absorbing state, *P*_*H*_, at increasing per-bit mutation probability (*μ*_*b*_). The main panel shows five overlapped curves for different population sizes *N*. The inset displays the same results for five larger values of *N*. Here we have overlapped the data for *N* = 500 shown in the main panel (black points indicated with the small arrow). Each data point for each curve is the value of *P*_*H*_ obtained from 10^3^ different equidistant initial conditions within the simplex Ω projected in (*S*_0_, *S*_4_) considering a single run for each initial condition. (**b**–**e**) Values of *P*_*H*_ obtained for different initial conditions within the simplex (*S*_0_, *S*_4_) using *N* = 500 and: (**b**) *μ*_*b*_ = 0.19; (**c**) *μ*_*b*_ = 0.2; (**d**) *μ*_*b*_ = 0.21; (**e**) *μ*_*b*_ = 0.22. Here each data point within the simplex is the mean value of *P*_*H*_ averaged over 100 independent replicas.

A more detailed characterization of the smooth nature of the transition responsible for the change from the tumor to the healthy state is displayed in Fig. 3a. Specifically, we have also computed *P*_*H*_ at increasing the per-bit mutation rate for several population sizes. The probability *P*_*H*_ has been computed slightly differently on this occasion. Now *P*_*H*_ is computed by dividing the number of initial conditions within the projection (*H*(*τ*), *S*_0_(*τ*)) achieving *H*^*as*^ by the total number of runs, which are given by a battery of 10^4^ equidistant initial conditions within this projection. We emphasize that for this computation of *P*_*H*_ a single run has been used for each initial condition. This way of computing *P*_*H*_ provides a more global information since we are sampling more points in the simplex Ω. The structure of the population for this computation has been fixed following the pattern of initial conditions within the projection (*H*(*τ*), *S*_4_(*τ*)) and filling the remaining population with sequences 010 and 110 at random. The results reveal that between the approximate range $0.16\lesssim {\mu }_{b}\lesssim 0.24$ some trajectories will reach the healthy state while some others will reach the tumor state, indicating the bistable nature of the dynamics. The shape of *P*_*H*_ at increasing *μ*_*b*_, close to a sigmoidal, remains similar for different values of *N* (Fig. 3a).

As mentioned, *P*_*H*_ has been previously computed from a battery of single initial conditions (1 replicate for each initial condition). However, since the dynamics is stochastic, different runs for the same initial condition could reach different asymptotic states. In order to check this possibility, we have computed the value of *P*_*H*_ within the projection (*H*(*τ*), *S*_4_(*τ*)). Here, as a difference from Fig. 3a, we have computed *P*_*H*_ for each one of the 10^4^ initial conditions. For each initial condition we have divided the number of trajectories achieving *H*^*as*^ by the total number of runs given by 100 replicates for each initial condition. Due to the large computational cost of running these analyses, we have restricted our analyses to *N* = 500. Specifically, we have performed these analyses for: *μ*_*b*_ = 0.19 (Fig. 3b); *μ*_*b*_ = 0.2 (Fig. 3c); *μ*_*b*_ = 0.21 (Fig. 3d); and *μ*_*b*_ = 0.22 (Fig. 3e). As expected, the probability of achieving the healthy state largely increases for initial conditions close to down-right corner of the simplex, where the population is started with a large number of healthy cells. As mutation rate increases, this region enlarges. Notice that for *μ*_*b*_ = 0.19 the region where *P*_*H*_ is close to one is very small and restricted to this corner in Ω.

The smooth transition characterized in Fig. 3a has been obtained using a particular set of initial conditions. In order to check the robustness of this smoothness upon the initial populations we have repeated the same analyses of Fig. 3 choosing the initial conditions from different regions of the simplex Ω. The same type of transition has been found sampling the projections (*H*(*τ*), *S*_2_(*τ*)) and (*H*(*τ*), *S*_6_(*τ*)), which are displayed in Fig. S3, and also follow a sigmoidal shape. Indeed, Fig. S3c reproduces similar sigmoidal curves by setting the initial populations completely at random. The simplexes displayed in panels a1,a2 and b1,b2 in Fig. S3 show the values of *P*_*H*_ computed within the simplex by using 100 runs for each initial population value. The cases a1 and a2 correspond to the simulations performed on the projection (*H*(*τ*), *S*_2_(*τ*)). Here, the remaining initial population of cells has been set by randomly choosing sequences 100 and 110. Alternatively, in panels b1 and b2 the remaining initial populations have been randomly chosen from sequences 100 and 010.

### Transitory times near bifurcation threshold

Typically, transients slow down as bifurcation values are approached. This can be understood from the ODEs model since the eigenvalues approach zero near bifurcation and thus a slower dynamics takes place: this involves longer transients. For example, the so-called critical slowing down, which appears near pitchfork and transcritical bifurcations, or the so-called delayed transitions, found near saddle-node bifurcations^54^. The mathematical model studied in^51^ allowed the analysis of transient times near bifurcation thresholds. Specifically, the time that a given initial condition spent to achieve the fixed point ${P}_{3}^{\ast }$, corresponding to the extinction of healthy cells, increased as *μ* → *μ*_*c*_, diverging at the critical bifurcation value. The same phenomenon was found for the dominance of the healthy cell populations as *μ* → *μ*_*c*_ from above.

**Figure 4:**
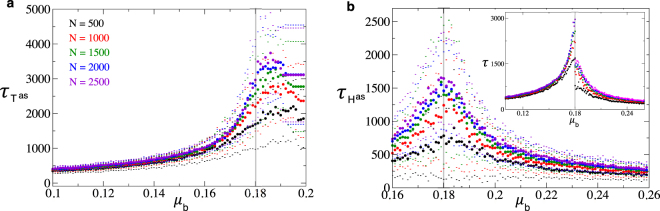
Mean times (in number of generations *τ*) needed for trajectories starting at *S*_0_(*τ* = 0) = 0.9 and *S*_4_(*τ* = 0) = 0.1 to achieve the tumor absorbing state *T*^*as*^ (**a**) and the healthy absorbing state *H*^*as*^ (**b**) at increasing the per-bit mutation probability. In both panels we plot the mean times represented with small circles (±SD, showing the upper and lower values) computed from 100 independent replicas achieving each absorbing state using five values of *N* (indicated inside panel (**a**)). The inset in (**b**) displays the time to *T*^*as *^below the transition and the time to *H*^*as*^ after the transition, putting together the data values of panels (a) and (b) (for the sake of clarity, the error values are not represented in the inset).

The stochastic simulations have been used to compute these times increasing the per-bit mutation probability towards its critical value. Since different absorbing states can be achieved from the same initial condition under the same probability (parameter) values, we have computed the mean times to each absorbing state following the next strategy. First, we have set the initial condition at (*H*(*τ* = 0), *S*_4_(*τ* = 0)) = (0.9, 0.1). Then, for each value of *μ*_*b*_ we have run different replicates of the same initial condition, discarding those trajectories reaching the other absorbing state. That is, for *μ* < *μ*_*c*_ we have discarded those trajectories reaching *H*^*as*^ (see Fig. 4a). Then, we have used the first 100 trajectories achieving *T*^*as*^ to compute the mean times to this absorbing state. The same strategy have been used to compute the times needed to achieve *H*^*as*^ after the transition. Since the critical value is not accurately identified, we have used the range 0.1 ≤ *μ*_*c*_ ≤ 0.2 in panel (a) of Fig. 4; and 0.16 ≤ *μ*_*c*_ ≤ 0.26 in panel (b) of Fig. 4. Interestingly, the time to *T*^*as*^ increases as *μ*_*b*_ increases, and this increase is much more pronounced for large population sizes. The same phenomenon is found above the bifurcation (Fig. 4b).

We note that below the bifurcation, the transient times increase up to *μ*_*b*_ ≈ 0.18, then becoming flat. This could be an indicator that the bifurcation values are around *μ*_*b*_ ≈ 0.18, as discussed. This is more clearly seen in panel (b) in Fig. 4, where the maximum time needed to achieve the healthy absorbing states is placed around *μ*_*b*_ ≈ 0.18. Since the system displays noise-induced bistability, it is possible to achieve either the healthy or the tumor absorbing state below and above the transition, respectively.

In order to provide more global information on the behavior of transients, we have computed the mean transient times dependence on the initial conditions. Here we have also computed these times in the projections (*H*(*τ* = 0), *S*_4_(*τ* = 0)). For those cases in which *H*(*τ* = 0) + *S*_2_(*τ* = 0) < 1, we have randomly initialized the rest of the simplex with sequences 110 and 010. Figure S4 displays these dependences of transients towards the healthy (upper row) and the tumor (lower row) absorbing states for three different population sizes (*N* = 500 (a), *N* = 1000 (b), and *N* = 1500 (c)) with *μ*_*b*_ = 0.21. Here, for each initial condition we have run 100 replicates, which have been used to obtain the mean transient times needed to reach each of the two absorbing states. The bigger simplexes in Fig. S4 show the mean times and the smaller ones display the associated standard deviations. Notice that variability is extremely large. The mean times for other population sizes have been computed, obtaining similar simplex patterns to those of Fig. S4 (results not shown). Similarly to what is observed in Fig. S4, the mean transients times increase with the size of the populations, for both times to *H*^*as*^ and to *T*^*as*^. For instance, the longest time to achieve *H*^*as*^ for *N* = 2000 was *τ* = 6352; *τ* = 6975 for *N* = 2500; and *τ* = 10245 for *N* = 5000. Similarly, for the longest times towards *T*^*as*^ we have found *τ* = 5932 for *N* = 2000; *τ* = 6334 for *N* = 2500; and *τ* = 12740 for *N* = 5000.

## Discussion

Tumors exhibit remarkable intracellular heterogeneity. Cellular heterogeneity often produces sub-populations of cancer cells able to survive to anticancer therapy, often leading to tumor relapses^55–58^. Such a heterogeneity has been suggested to play a key role in the fate of cancer cells, providing a selective advantage in the face of drug administration and tumor cells adaptation. Genome instability has been suggested as one of the main engines responsible for the high variability and heterogeneity of tumor cells, mainly generated through chromosome and microsatellite instabilities, involving extremely large mutation rates and the emergence of the so-called mutator phenotypes^11,13,15^. These properties have been studied using the quasispecies framework^21–25^, largely applied for RNA viruses due to the high mutation rates of these pathogens.

In this manuscript we have performed stochastic simulations of a dynamical system composed of tumor cells with different phenotypes that compete with healthy cells. In a recent article^51^ we provided a detailed description of the deterministic dynamics for this system using Eigen’s quasispecies equation. This mean field model revealed two possible asymptotic states given by dominance and extinction of tumor cells. The change between these two asymptotically globally stable states was determined by the so-called trans-heteroclinic bifurcation (see also ref.^52^ for a detailed description of this bifurcation). As mentioned, the mean field model discarded bistable scenarios below and above the bifurcation. Particularly, the two fixed points corresponding to these two asymptotic states are connected heteroclinically, only one of them being stable, depending on the replication and mutation parameters. Here we describe a novel mechanism of noise-induced bistability, for which stochastic fluctuations due to finite size populations give place to bistable dynamical scenarios in which either one of these two asymptotic states can be reached under the same parameter values and same initial conditions.

It is known that random fluctuations can generate novel behavior in dynamical systems, for instance, noise-enhanced stabilization^59,60^, stochastic resonance^61^, or the so-called noise-induced transitions^62,63^. However, more recent discoveries have characterized the emergence of noise-induced bistability in systems that are monostable in their deterministic limit. This noise-induced bistability has been recently described in stochastic simulations for a simple catalytic loop scheme^64^. The mean field model for this system revealed a unique attractor within phase space. More recently, this phenomenon has also been described in stochastic models in foraging ant colonies (see^65^ and references therein). Noise-induced bistability was also found experimentally in positive transcriptional feedback loops not displaying bistability in the continuum, deterministic limit^66^.

The phenomenon described in this manuscript giving place to noise-induced bistability is different from the ones mentioned above, since for our case the mean field model indicates the presence of two fixed points placed at the boundaries of the phase space. However, these fixed points have an opposite stability character (i.e., one being stable and the other unstable), thus no possible bistability is found in the deterministic approach^51^. As we show here, noise due to finite size effects makes the presence of bistability possible under this scenario. The phenomenon described in our manuscript can be also interpreted as a kind of noise-enhanced stability (NES) typical from metastable systems (see refs^46,67^ for examples of NES on other cancer stochastic systems). This is a resonance-like behavior that indicates that the average lifetime of a particle in the metastable state can be enhanced with respect to the deterministic approach^68,69^. It is known that for a classical Brownian particle in a metastable (cubic) potential, the average time as a function of noise intensity has a maximum when the particle is initially placed outside a stable state. This noise-induced phenomenon has been identified in superconductor physics^70^ and in quantum systems^71^. We must note, however, that our system is not (strictly speaking) metastable, since there is a single stable state when the system is not at the bifurcation point. The stabilization of the dynamics in our model is due to the multiplicative nature of the noise^72^ tied to finite size populations, which drives trajectories towards the absorbing states.

Our simulations considered intrinsic (demographic) noise. However, one might expect the emergence of noise-induced bistability as well under extrinsic noise, since external fluctuations could also allow trajectories to achieve any of the absorbing states of the system, although the probabilities of achieving absorbing states and the transients involved could be different depending on whether the noise is intrinsic or extrinsic, probably also depending on the color of the noise for non-Gaussian noise. The growth rate of tumors can also be affected by many environmental factors. For instance, random variations in parameters tied to nutrients and oxygen supply, degree of vascularization of tissues, chemical agents or radiations under therapeutic treatments, among others (see ref. ^47^ and references therein).

The mean field approach for the system explored in this manuscript revealed a sharp and discontinuous transition responsible for tumor clearance^51^. Here we have shown that demographic fluctuations can break this catastrophic behavior, and, even when the bifurcation values are surpassed, the probability of achieving the healthy state remains low and increases monotonously. This means that stochastic fluctuations could impair the extinction of tumor cells even once mutation rates are highly pernicious for tumor cells. Finally, we must note that our models assume that healthy cells cannot change to a tumor phenotype. This is a strong assumption that may be plausible over short time scales. Our models reveal that the transition from tumor to healthy cells can be achieved by increases in mutation rates or in the accumulation or genome anomalies during replication. However, long exposures to radiation or to chemotherapies could also involve transitions from healthy to tumor cells^73^. In terms of our modeling approach this would involve considering a mutation rate for healthy cells, and the absorbing nature of the healthy state would be broken. Future research should consider this new scenario both in the mean field model and in stochastic versions. This approach might offer a good modeling framework to investigate how the trans-heteroclinic transition changes by coupling the healthy cells state with the tumor cell phenotypes within sequence space by diffusion (mutation), also offering an opportunity to investigate the dynamics of tumor relapses during long therapeutics or high chemotherapeutic dosage^73,74^.

## Methods

### Stochastic bit-strings model

To explore the role of stochasticity in the dynamics and the transitions of phenotypic cancer quasispecies an agent-based probabilistic model is built, in which cell phenotypic traits are coded by means of bit-strings. Bit-strings simulation models are useful tools to investigate *in silico* evolution, and have been employed to charaterize the evolutionary dynamics of RNA viruses^75^, cancer quasispecies^21,25^, and genetic algorithms^76,77^, among others. Our computational model is implemented with a MonteCarlo method and considers a constant population of *N* cells, each cell named as ${S}_{abc}^{(i)}$, with *i* = 1, ..., *N*, and *a*, *b*, *c* ∈ {0, 1} i.e., each of them having a phenotype encoded in a bits string of length *ν* = 3 bits, indicated in the subindex of *S*. As mentioned, each bit corresponds to a given compartment: 1*st* bit: replication-related genes compartment; 2*nd* bit: genes responsible for genome integrity; and 3*rd* bit: *hk* genes compartment. Anomalies in any of these compartments are coded by bit 1. Hence, the healthy state is coded by sequence 000 (see Fig. S1).

The simulation algorithm works as follows (see Fig. S1(c)): at each time generation, *τ*, we randomly choose *N* cells of the population. Every time we choose a random cell, named ${S}_{abc}^{(j)}$, we apply the rule of replication. This asynchronous updating ensures that (on average) all cells will be updated once per generation. The rule of replication is implemented by randomly choosing a different cell of the population, say ${S}_{abc}^{(k)}$ with *k* ≠ *j*. Depending on the phenotype of cell ${S}_{abc}^{(j)}$, the following reactions are implemented with probabilistic rates. Healthy cells reproduce with probability *r* ∈ [0, 1]. Following^51^ we assume that the mutation rate of healthy cells is negligible compared to the mutation rate of tumor cells. Hence, healthy cells reproduce following the next reaction (blue arrows in Fig. S1(c)):

$${S}_{000}^{(j)}+{S}_{abc}^{(k)}\,\mathop{\longrightarrow }\limits^{r}\,{S}_{000}^{(j)}+{S}_{000}^{(k)},$$

Cancer cells can proliferate with error-free replication (solid red arrows in Fig. S1(c)) or making anomalous copies of themselves (dashed red arrows in Fig. S1(c)). The error-free reaction of the cells with sequence *S*_100_ is:

$${S}_{100}^{(j)}+{S}_{abc}^{(k)}\,\mathop{\longrightarrow }\limits^{(r+{\delta }_{r}\mathrm{)(1}-{\mu }_{b}{)}^{\nu }}\,{S}_{100}^{(j)}+{S}_{100}^{(k)}\mathrm{.}$$

Since cells with state 100 have anomalies in the replication-related genes, they reproduce with probability (*r* + *δ*_*r*_) ∈ [0, 1], *δ*_*r*_ being the increase in proliferation rates tied to the anomalies found in the replication compartment. Here *μ*_*b*_ ∈ [0, 1] is the per-bit mutation probability of tumor cells and *ν* is the length of the string (as mentioned, here with *ν* = 3). Probability *μ*_*b*_ can also be considered as a probabilistic rate at which cells accumulate damage at each compartment (by means of e.g., gene loss, chromosomal breaks). The reaction for the erroneous replication of cells with this sequence is:

$${S}_{100}^{(j)}+{S}_{abc}^{(k)}\,\mathop{\longrightarrow }\limits^{(r+{\delta }_{r}\mathrm{)(1}-{\mu }_{b}{)}^{\nu -1}{({\mu }_{b})}^{\nu -2}}\,{S}_{100}^{(j)}+{S}_{1bc}^{(k)},$$

here with *b* = 1 and *c* = 0, or *b* = 0 and *c* = 1 (recall that here no backward mutations are allowed, following^51^). For cells with state 010 the reaction of error-free reproduction reads:

$${S}_{010}^{(j)}+{S}_{abc}^{(k)}\,\mathop{\longrightarrow }\limits^{r{\mathrm{(1}-({\mu }_{b}+{\delta }_{\mu }))}^{\nu }}\,{S}_{010}^{(j)}+{S}_{010}^{(k)},$$

while the erroneous reproduction is given by:

$${S}_{010}^{(j)}+{S}_{abc}^{(k)}\,\mathop{\longrightarrow }\limits^{r{\mathrm{(1}-({\mu }_{b}+{\delta }_{\mu }))}^{\nu -1}{({\mu }_{b}+{\delta }_{\mu })}^{\nu -2}}\,{S}_{010}^{(j)}+{S}_{p1m}^{(k)},$$

with *p* = 1 and *m* = 0 or *p* = 0 and *m* = 1. Here \delta_*μ*_ is the increase in mutation probabilities due to the anomalies tied to the compartment of genome instability. The other reproducing tumor cells have state 110. These cells have anomalies in both replication-related and stability compartments. Their error-free reproduction is:

$${S}_{110}^{(j)}+{S}_{abc}^{(k)}\,\mathop{\longrightarrow }\limits^{(r+{\delta }_{r}\mathrm{)(1}-({\mu }_{b}+{\delta }_{\mu }{))}^{\nu }}\,{S}_{110}^{(j)}+{S}_{110}^{(k)},$$

the reaction of erroneous reproduction being:

$${S}_{110}^{(j)}+{S}_{abc}^{(k)}\,\mathop{\longrightarrow }\limits^{(r+{\delta }_{r}\mathrm{)(1}-({\mu }_{b}+{\delta }_{\mu }{))}^{\nu -1}{({\mu }_{b}+{\delta }_{\mu })}^{\nu -2}}\,{S}_{110}^{(j)}+{S}_{111}^{(k)}\mathrm{.}$$

Finally, the cells with anomalies in the *hk* genes compartment do not reproduce i.e., *S*_*ab*1_, with *a*, *b *∈ {0, 1}. For the sake of simplicity, we hereafter will label the cells with a subindex given by the integer number corresponding to the binary sequence. That is, we will consider: ${S}_{0}^{(j)}={S}_{000}^{(j)}$, ${S}_{1}^{(j)}={S}_{001}^{(j)}$, ${S}_{2}^{(j)}={S}_{010}^{(j)}$, …, ${S}_{7}^{(j)}={S}_{111}^{(j)}$ (see Table SI). Moreover, we will also define the fraction of healthy cells (normalized population) as

$$H=\frac{1}{N}\sum _{i=1}^{N}{S}_{0}^{(i)}\mathrm{.}$$

Generally, we will hereafter talk about the healthy absorbing state, *H*^*as*^, as an equilibrium state where the whole population is composed by healthy cells (*S*_0_). This state corresponds to the fixed point ${P}_{2}^{\ast }$ identified with the mean field model in ref.^51^. (see Section S1.B. in the Supplementary Material, SM). Assuming a normalized population, it is clear that the total number of tumor cells, labeled *T*, is *T* = 1 − *H*, or, alternatively

$$T=\frac{1}{N}\sum _{i=1}^{N}\sum _{j=1}^{7}{S}_{j}^{(i)}\mathrm{.}$$

Here we also define the tumor absorbing state, *T*^*as*^, which involves a stationary population without healthy cells, corresponding to the fixed point ${P}_{3}^{\ast }$ in^51^ (see Section 1B in the SM for the discussion on the absorbing states in the context of the stochastic model).

The normalization of the populations allows us to define a state space for this stochastic dynamical system given by an eight-dimensional simplex:

$${\rm{\Omega }}:=\,\{{S}_{j=\mathrm{0,...7}}\in {{\mathbb{R}}}^{8}|{S}_{j}\ge \mathrm{0,}\,\frac{1}{N}\sum _{i=1}^{N}\sum _{j=0}^{7}{S}_{j}^{(i)}=\mathrm{1\}.}$$

The dynamics in terms of population asymptotic states as well as in transients will be mainly analyzed using projections of Ω for several initial conditions or for a single initial populaiton. If not otherwise specified, we will consider as initial conditions a population given by a 90% of healthy cells and a 10% of tumor cells with driver mutations (anomalies in compartment *R*) i.e., *S*_0_(*τ* = 0) = 0.9, *S*_4_(*τ* = 0) = 0.1, and thus *S*_*k*_(*τ* = 0) = 0, *k* = 1, 2, 3, 5, 6, 7, as initial conditions. These initial conditions assume that a small number of tumor cells initiate the process of tumorigenesis, like in reality. However, as mentioned, other initial populations will be explored.

## Electronic supplementary material

Supplementary material
