# Supplementary material for: Noise-induced bistability in the fate of cancer phenotypic quasispecies: a bit-strings approach
Source: Sci Rep. 2018 Jan 18;8:1027. doi: 10.1038/s41598-018-19552-2 (PMC5773630; doi:10.1038/s41598-018-19552-2)
Supplement: Supplementary file 1 — Supplementary material [file 41598_2018_19552_MOESM1_ESM.pdf]

Noise-induced bistability in the fate of cancer phenotypic quasispecies: a bit-strings approach

Supplementary Material

Josep Sardanyés[1,2,*] Tomás Alarcón[3,4,1,2]

1. Centre de Recerca Matemàtica, Campus de Bellaterra, Edifici C, 08193 Bellaterra, Barcelona, Spain

2. Barcelona Graduate School of Mathematics (BGSMath). Campus de Bellaterra, Edifici C, 08193 Bellaterra, Barcelona, Spain

3. ICREA, Pg. Lluis Companys 23, 08010 Barcelona, Spain

4. Departament de Matemàtiques, Universitat Autònoma de Barcelona, Barcelona, Spain

∗ corresponding author

CONTENTS

I. Phenotypic cancer quasispecies 1

A. Differential equations model 1

B. Fixed points and absorbing states 4

References 4

I. PHENOTYPIC CANCER QUASISPECIES

In this section we describe the mathematical model based on ordinary differential equations (ODEs) used to investigate the deterministic dynamics of the dynamical system explored in this article. We also provide a summary of the main results derived from the ODEs model.

A. Differential equations model

Recently, a differential equations model describing the population dynamics of healthy cells competing with a heterogeneous pool of cancer cells phenotypes has been investigated in Ref. [1]. This mathematical model, based on Eigen's quasispecies equation [2], considered a well-mixed and constant population of healthy cells competing with tumor cells carrying mutations or

anomalies in both tumor-suppressor genes and proto-oncogenes ($1st$ bit or compartment), in genes responsible for genomic integrity ($2nd$ bit) and in house-keeping (hk) genes ($3rd$ bit). These three compartments were defined as bits in this minimal system giving place to different tumor cell phenotypes competing with healthy cells, given by strings 000. The full system, corresponding to the so-called quasispecies-like phenotypic models (see also [3]), describes the dynamics of different cancer cell phenotypes with a quasispecies populational structure (see Fig. S1). The characteristics of this population are the same as the ones displayed in Table I for the agent-based model investigated in this article.

The dynamical system describing the deterministic dynamics of this system is given by [1]:

$$\dot{x}_{000} = \frac{dx_0}{dt} = rx_0 - x_0\Phi, \quad (1)$$

$$\dot{x}_{001} = \frac{dx_1}{dt} = -x_1\Phi, \quad (2)$$

$$\dot{x}_{010} = \frac{dx_2}{dt} = (1-(\mu+\delta_\mu))rx_2 - x_2\Phi, \quad (3)$$

$$\dot{x}_{011} = \frac{dx_3}{dt} = \frac{1}{\nu-1}(\mu+\delta_\mu)rx_2 - x_3\Phi, \quad (4)$$

$$\dot{x}_{100} = \frac{dx_4}{dt} = (1-\mu)(r+\delta_r)x_4 - x_4\Phi, \quad (5)$$

$$\dot{x}_{101} = \frac{dx_5}{dt} = \frac{1}{\nu-1}\mu(r+\delta_r)x_4 - x_5\Phi, \quad (6)$$

$$\dot{x}_{110} = \frac{dx_6}{dt} = (1-(\mu+\delta_\mu))(r+\delta_r)x_6 + \frac{1}{\nu-1}\Big[(\mu+\delta_\mu)rx_2 + \mu(r+\delta_r)x_4\Big] - x_6\Phi, \quad (7)$$

$$\dot{x}_{111} = \frac{dx_7}{dt} = (\mu+\delta_\mu)(r+\delta_r)x_6 - x_7\Phi, \quad (8)$$

where $\Phi = r(x_0 + x_2) + (r + \delta_r)(x_4 + x_6)$ is the dilution flow which introduces competition between all the cell populations, also keeping a constant population. The state variables $x_i(t)$, $i = 0, ..., 7$, are the relative concentration or population numbers of cells with sequences 000, ..., 111. As done for the stochastic bit-strings model, subinidices of the population variables correspond to the integer number of the binary sequences. The model parameters are given by the replication rate of cells ($r > 0$); the increase of proliferation of tumor cells ($\delta_r > 0$); the rate of mutation or accumulation of genomic anomalies of tumor cells $0 < \mu < 1$; and the increase of genome instability of tumor cells $0 < \delta_\mu < 1 - \mu$. That is, tumor cells with bit 1 in the first position of the string present increased proliferation rates, $r + \delta_r$, due to mutations or anomalies in replication-related genes. Genome instability in introduced with $\mu + \delta_\mu$ when the second bit of the strings is 1. The cells with sequences $ab1$, $a, b \in \{0, 1\}$ present anomalies or mutations in hk genes and thus

are not able to proliferate. Finally, ν is the length of the sequences i.e., here with $\nu = 3$.

The fixed points of Eqs. (1)-(8) and their stability was characterized in [1]. Among the fixed points identified, two of them were responsible for the two asymptotic states behind tumor persistence and extinction. These two fixed points, labeled P_2^* and P_3^* in [1] are the ones displayed in Fig. S2. Interestingly, the asymptotic states identified with the MonteCarlo simulation model developed in this article correspond to these two equilibria (compare the projections in Ω of Figs. 1 and 2 in the main manuscript with the simplexes of Fig. S2). According to the convention adopted in this manuscript, the fixed point P_2^* corresponds to the healthy absorbing state H^{as}, while the fixed point P_3^* is the tumor absorbing state T^{as}.

According to Fig. S2, when $\mu < \mu_c$ the fixed point P_3^* is globally asymptotically stable while P_2^* is unstable. Recall that $\mu_c = \delta_r/(r + \delta_r)$. We note that these two fixed points present a heteroclinic connection (see below for the definition of heteroclinic connection). At the bifurcation value $\mu = \mu_c$ the heteroclinic connection is replaced by a line of fixed points. After the bifurcation, when $\mu > \mu_c$, the nature of the stability of these two fixed points has been reversed, P_2^* being asymptotically globally stable and P_3^* being unstable. Hence, the dynamics of the system under study under its mean field limit involves monostability when $\mu \neq \mu_c$. This means that the stable fixed points are globally stable and neither coexistence of solutions nor different basins of attraction can be found in the phase space of Eqs. (1)-(8) [1]. As mentioned, these two equilibrium points are connected heteroclinically. The so-called heteroclinic connection can be defined as follows:

Definition I.1 (Heteroclinic connection). *Let x_1^* and x_2^* be equilibria of a nonlinear function $f : \mathbb{R} \to \mathbb{R}^n$:*

$$\dot{x}(t) = f(x(t)), \qquad (9)$$
$$\text{s.t.} \quad \mathrm{x}(0) = x_0 \in \mathbb{R}^n,$$

where f is continuously differentiable. An orbit $\mathrm{Or}(x_0)$ starting at a point $x_0 \in \mathbb{R}^n$ is called a heteroclinic connection of x_1^* and x_2^* if $\lim_{t\to-\infty} \phi^t(x_0) = x_1^*$ and $\lim_{t\to\infty} \phi^t(x_0) = x_2^*$, $\phi^t(x_0)$ being the value of a trajectory starting at the point x_0 at time t.

Remark I.1. *From Definition II.1 it immediately follows that the heteroclinic connection is a part of the unstable manifold of x_1^*, $W^u(x_1^*)$, as well as of the stable manifold of x_2^*, $W^s(x_2^*)$.*

B. Fixed points and absorbing states

As mentioned above, the stability of the fixed points P_2^* and P_3^* depends on the parameters μ, r, and δ_r (see [1] for further details). It is important to note that these fixed points are absorbing states, regardless of their stability. It means that once one of these two fixed points is achieved by a trajectory, the system will remain trapped in that fixed point forever. For the deterministic model, the fixed points that the orbits will reach correspond to the stable ones whenever the initial condition does not coincide with the coordinates of the fixed point. However, in the stochastic model, noise can make trajectories to reach a fixed point that in the deterministic model is unstable. Since these are absorbing states, the system will get trapped in one of these two fixed points reached by a stochastic trajectory, remaining there forever. The absorbing nature of these two fixed points becomes clear from the structure of the population and from the dynamical processes under consideration. Two important features determine the absorbing nature of these two equilibria: healthy cells do not mutate and backward mutations are not allowed. This means that once healthy cells become extinct, no possible production of S_0 is possible from the pool of mutants (since no backward mutations are allowed). Similarly, once the entire population is formed by healthy cells, no possible production of mutants is possible since S_0 cells cannot produce mutants.

[1] Sardanyés J, Martínez R, Simó C, Solé RV. Abrupt transitions to tumor extinction: a phenotypic quasispecies model. J. Math. Biol. 74(7):1589-1609 (2017)

[2] Eigen, M. Selforganization of Matter and the Evolution of Biological Macromolecules. *Die Naturwiss.* **58**, 465 (1971).

[3] Sardanyés J, Simó C, Martínez R, Solé RV, Elena SF. Variability in mutational fitness effects prevents full lethal transitions in large quasispecies populations. Sci. Rep. 4:4625 (2014)

Supplementary figures and tables

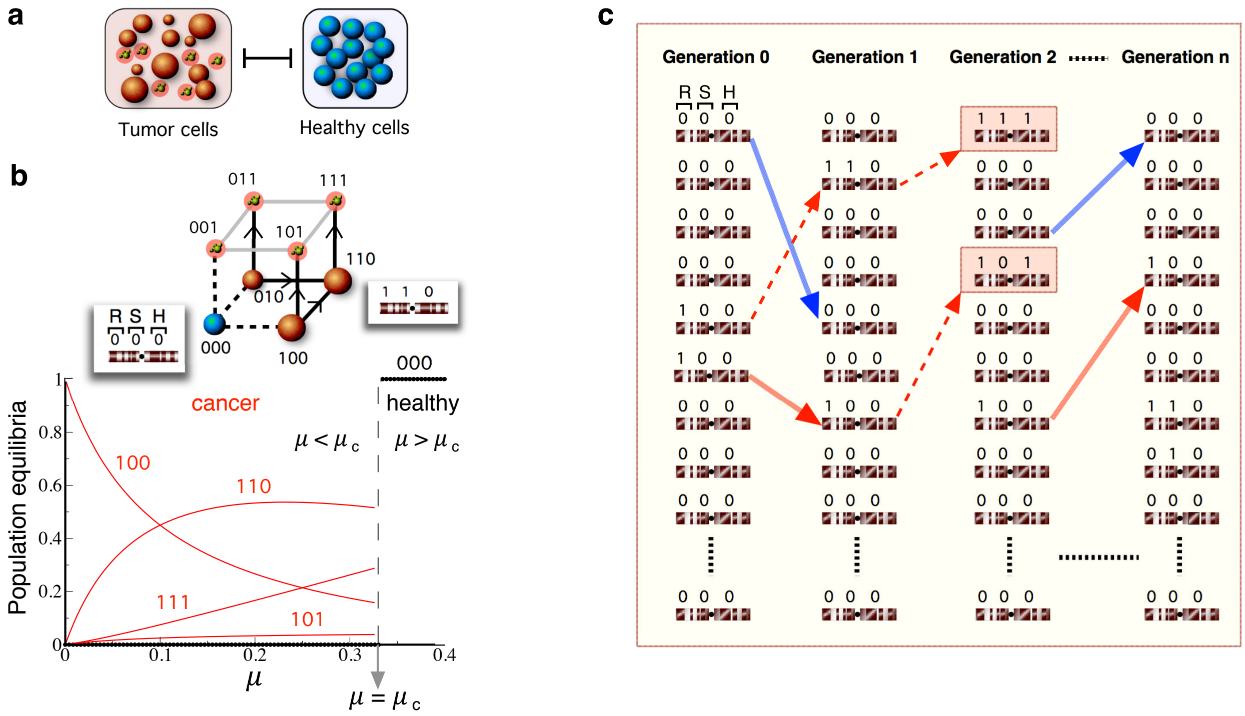

FIG. 1. (a) Schematic diagram of the cancer quasispecies model, in which different tumor phenotypes compete with healthy cells (see [1]). (b) Sequence space for the cancer phenotypes and healthy cells (sequence 000). Here tumor phenotypes include cells with mutations or anomalies (denoted by bit 1) in replication-related genes (e.g., proto-oncogenes and tumor suppressor genes, compartment R); anomalies in genes preserving genome stablity (compartment S); and anomalies in hk genes (compartment H). Alterations in these compartments are assumed to define different cancerous phenotypes. The mean field model studied in [1] revealed a catastrophic transition governed by a trans-heteroclinic bifurcation when increasing the mutation rate of tumor cells above a critical threshold μ_c. This bifurcation, displayed below the sequence space in (b), separates two different scenarios: persistence of tumor cells and vanishing of the healthy ones when $\mu < \mu_c$ and extinction of tumor cells and dominance of healthy cells when $\mu > \mu_c$. (c) Diagram of the bit-strings stochastic model used to simulate the dynamics of cancer phenotypic quasispecies considering a finite population of cells. Here blue arrows indicate the replication of healthy cells along generations. Red arrows indicate the replication of tumor cells, which can be accurate (solid arrows) or error-prone (dashed arrows) giving place to other tumor phenotypes. Sequences inside the red boxes do not replicate since they present anomalies in the hk genes.

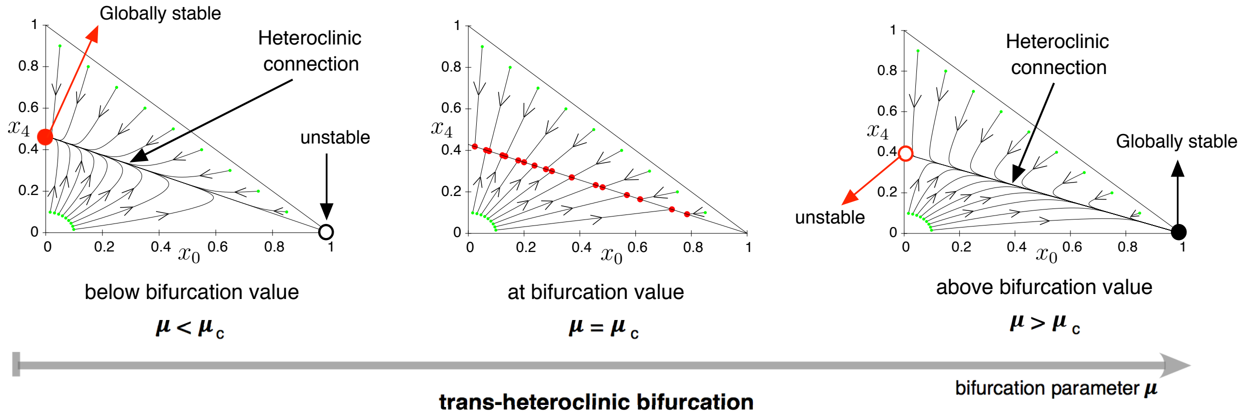

FIG. 2. Phase portraits for the ODEs model analyzed in [1], computed numerically using $r = 0.1$, and $\delta_r = \delta_\mu = 0.05$. Three qualitatively different scenarios are displayed: (left simplex) below the bifurcation the only stable fixed point corresponds to the dominance of the tumor cell phenotypes and the extinction of healthy cells (fixed point P_3^* in [1]). At the bifurcation value the heteroclinic connection is replaced by a line of fixed points (mid panel). Above the bifurcation, the only stable fixed point corresponds to the healthy state (fixed point P_2^* in [1]), where tumor cell phenotypes become extinguished (right panel). The fixed points P_3^* and P_2^* are asymptotically globally stable below and above the bifurcation value, respectively. That is, before and after the trans-heteroclinic bifurcation the system is monostable.

Cells		Phenotype	
State (sequence)	Subindex	Replication	Per-bit mutation
S_{000}	S_0	r	0
S_{001}	S_1	0	–
S_{010}	S_2	r	$\mu_b + \delta_\mu$
S_{011}	S_3	0	–
S_{100}	S_4	$r + \delta_r$	μ_b
S_{101}	S_5	0	–
S_{110}	S_6	$r + \delta_r$	$\mu_b + \delta_\mu$
S_{111}	S_7	0	–

TABLE I. Quasispecies-like population simulated with an *in silico* stochastic model considering a population of cells with three compartments corresponding to replication-related genes (first bit); to genes responsible for genomic integrity (second bit); and to house-keeping (hk) genes (third bit). Those cells with sequence S_{000} do not have mutations or anomalies in any of the three compartments and are considered as cells with the healthy phenotype. All the other sequences with some anomaly or mutation (given by bit 1) in any of the compartments are considered as tumor cells. The table shows the binary code for each cell (first column) and the subindex used for each cell (second column). The third and fourth columns indicate the phenotype of each cell, given by both replication and per-bit mutation probabilities.

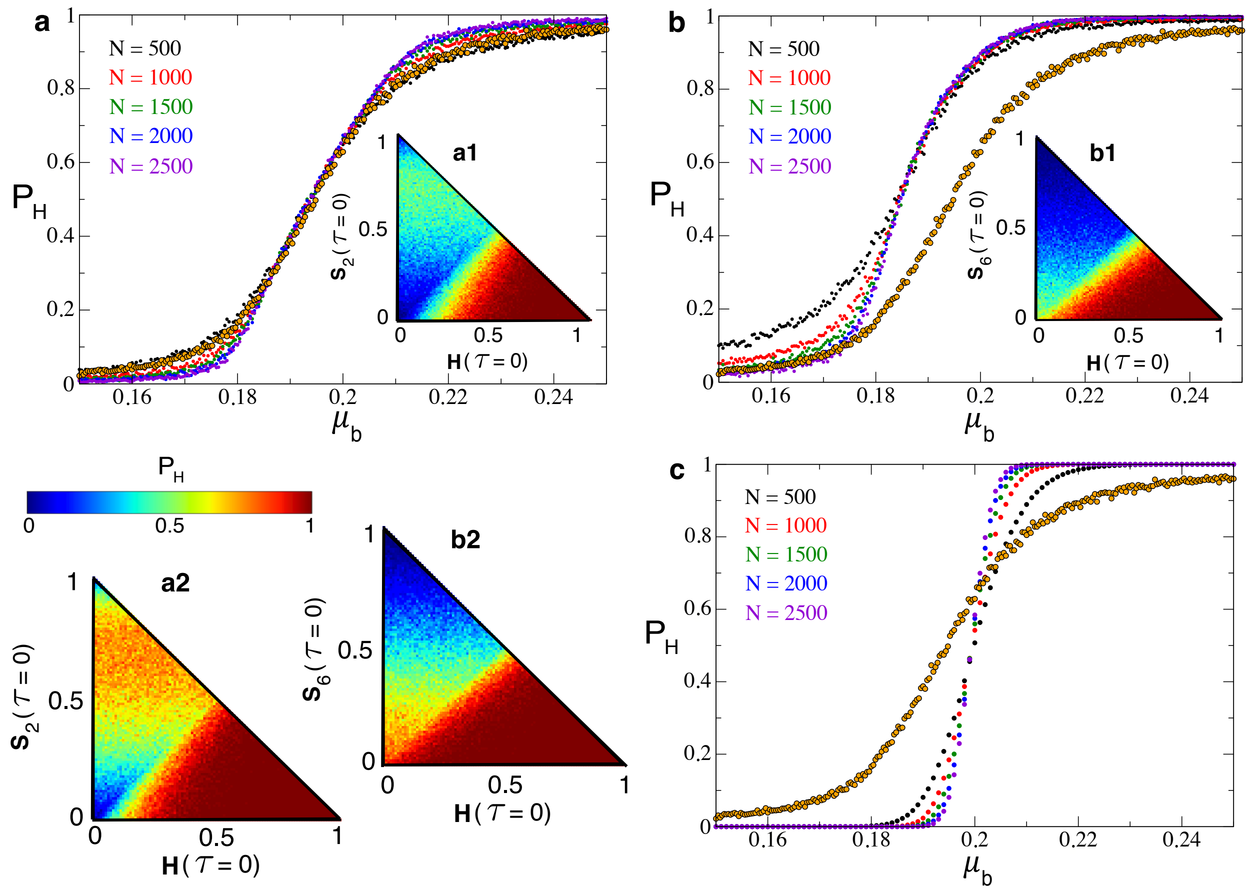

FIG. 3. Probability of achieving the healthy equilibrium state, P_H, at increasing the per-bit mutation rate (μ_b) under different initial conditions and for different population sizes N. Each data point for each curve in panels (a-c) is the value of P_H obtained from 10^3 different equidistant initial conditions within the simplex (S_0, S_2) (a); and within the simplex (S_0, S_6) (b). Panel (c) displays the same results for P_H computed from 10^5 different random initial conditions within the eight-dimensional simplex. In panels (a-c) we show overlapped the value of P_H obtained from the projection (S_0, S_4) displayed in Fig. 3a in the main manuscript (data displayed with bigger orange circles). Two pairs of simplexes are displayed: $\mu_b = 0.21$ (inset a1 in panel (a) and inset b1 in panel (b)); and $\mu_b = 0.22$ (a2 and b2). The values of P_H represented within the simplexes have been also computed averaging over 100 independent replicas for each set of initial conditions.

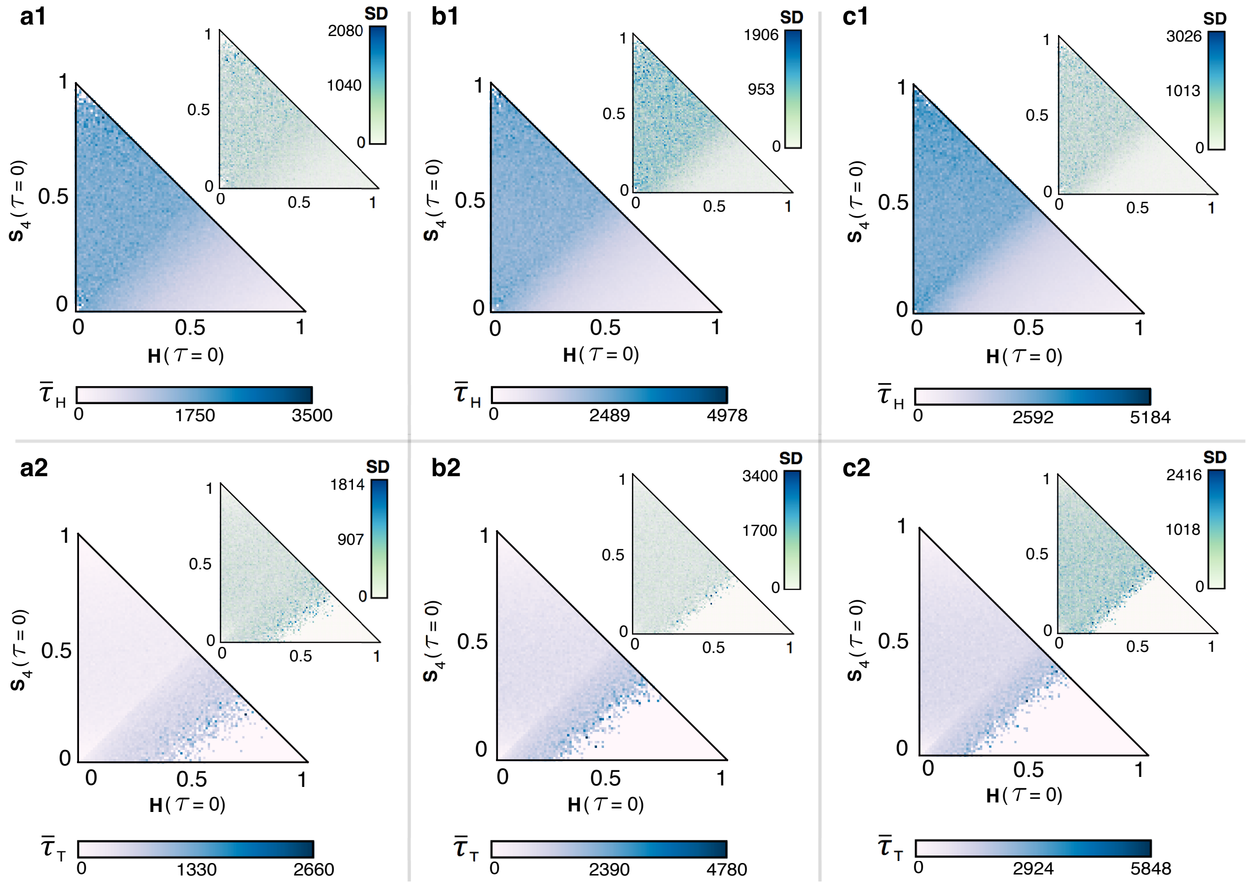

FIG. 4. Dependence of the mean times the stochastic trajectories spend to achieve each of the absorbing states H^{as} or T^{as} with the initial conditions. We display the results setting $\mu_b = 0.21$ for different population sizes using: (a) $N = 500$; (b) $N = 1000$; and (c) $N = 1500$. The first row (indicated with labels 1) displays the mean times (bigger projected simplexes) the trajectories need to achieve the absorbing state H^{as}. The smaller projections of the simplex display the standard deviations. The second row (label 2) shows the same results for transients towards T^{as}. For each initial condition we have computed the mean times and the standard deviations (SD) from 100 independent replicas.
